# Integrating drivers of pro-environmental behavior and physical activity to explore (in) compatibilities between an active and an environmentally sustainable lifestyle

**DOI:** 10.3389/fpsyg.2024.1397320

**Published:** 2024-12-11

**Authors:** Louise Eriksson, Stefan Linde

**Affiliations:** ^1^Department of Economics, Geography, Law and Tourism, Mid Sweden University, Östersund, Sweden; ^2^Department of Geography, Umeå University, Umeå, Sweden; ^3^Department of Humanities and Social Sciences, Mid Sweden University, Östersund, Sweden

**Keywords:** travel mode choice, secondhand purchase, identity, motivation, sport and outdoor

## Abstract

**Introduction:**

Sport and outdoor activities have benefits on people’s health and well-being but may also increase the frequency of unsustainable behaviors. The present study explores drivers of travel mode choice and consumption of material (clothes and equipment) associated with physical activity to clarify the extent to which an active and sustainable lifestyle is compatible. The role of identity and varying levels of internalized motivation for pro-environmental behaviors (autonomous and controlled environmental motivation) and engagement in physical activity (autonomous and controlled activity motivation) was examined. In addition, socio-demographic, physical context, and life situation correlates of environmentally significant behaviors associated with physical activity were analyzed.

**Methods:**

A survey of a random sample of the general public in Sweden (*n* = 1013) was conducted.

**Results:**

After controlling for hours of physical activity, the study showed that environmental self-identity was related to a lower likelihood of using the car alone via autonomous environmental motivation and to a higher likelihood of buying and selling used material via controlled environmental motivation. Physical activity drivers displayed diverse impacts on environmentally significant behaviors, e.g., athlete identity was associated with a higher likelihood of using the car alone and buying new material, but also selling used material. Being a member of a sport or outdoor organization was related to a higher likelihood of using the car alone and buying new material, but also using active travel modes as well as buying and selling used material.

**Discussion:**

With a better understanding of the drivers of environmentally significant behaviors in this domain, strategies to encourage sustainable transport and circular flows of material in sports and outdoors can be outlined.

## 1 Introduction

### 1.1 Environmentally significant behaviors in sports and outdoors

Participation in sports, nature recreation activities, and other forms of physical activity, is key for a healthy lifestyle (World Health Organization (WHO), 2020). However, these healthy lifestyle behaviors may come in conflict with the pursuit of a more environmentally sustainable lifestyle. For example, active sport participants have been found to have a higher carbon footprint from traveling compared to the population average (Wicker, 2019). Sport participants competing on a regional level or higher also display a higher carbon footprint from traveling to training, compared to those not competing and those competing locally (Thormann and Wicker, 2021). Research has also suggested that the performance logic, often dominant in sports, may at times conflict with principles of sustainable practices (Engström et al., 2018; Backman and Svensson, 2022). In contrast, other studies have discovered a weak positive association between outdoor recreation activities and some pro-environmental behaviors such as recycling, donating money, and participating in environmental groups (Theodori et al., 1998; Larson et al., 2011). However, as there are often practical obstacles to adopting sustainable practices when participating in outdoor recreation, such as a lack of access to public transportation (Juschten and Preyer, 2023), outdoor recreation is not necessarily associated with more sustainable behavior. Some have argued that spending more time outdoors may only result in a more sustainable lifestyle if it is paired with a conscious reflection on the human-nature relationship (Beery and Wolf-Watz, 2014; Høyem, 2020). How physical activity in various forms, inside and outdoors, as part of organized activities or individually, is associated with unsustainable lifestyles does, as such, warrant further investigation. One direction to explore this, is through studying the motivational factors relevant for environmentally significant behaviors in this domain, to expose compatibilities and incompatibilities between an environmentally sustainable and physically active lifestyle. While pro-environmental behavior refers to behaviors minimizing the harm to the environment (Larson et al., 2015), environmentally significant behaviors can be used as an umbrella term encompassing behaviors with a minor or greater impact on the environment (Stern, 2000).

Drawing on environmental psychology and sport psychology, we seek to understand how psychological drivers of pro-environmental behaviors in interaction with physical activity drivers may encourage, or discourage, environmentally significant behaviors associated with physical activity. Using a sample of the general public in Sweden, this study focuses on travel mode choice and purchase of clothes and equipment, representing behaviors enabling the engagement in physical activities. However, depending on the individual’s decisions, these behaviors may have negative implications on environmental sustainability. Although motivations to be physically active mostly have been considered as a driver of active travel modes such as cycling (Charreire et al., 2021; Renninger et al., 2022), there is an increasing interest in understanding the relationship between physical activity and environmental sustainability more generally (Hutchinson et al., 2015; Cunningham et al., 2020; Bernard et al., 2022). This is also where our research comes in.

### 1.2 Factors associated with environmentally significant lifestyle behaviors

Absolute reductions and shifting modes of consumption, but also increasing the longevity of products, and expanding sharing practices, are ways to increase the sustainability of travel behavior, energy use, consumption, and waste management (Sandberg, 2021). In general, environmentally significant lifestyle behaviors are influenced by contextual factors, available sustainable alternatives, socio-demographics (including resources), the situation, and a number of psychological factors (Stern, 2000).

Previous research has, for example, revealed that women drive less than men, that younger people use public transport and active travel modes more often than older people, and that households with children travel more by car than households without (Susilo et al., 2019; Kawgan-Kagan, 2020; Bi and Romao, 2021). Although leisure trips generally are understudied, one study focusing on exercise and outdoor trips found that having children in the household was associated with more milage by car, but that there was no gender difference, and only a slight difference between rural and urban areas (Strömblad et al., 2022). A recent review revealed that gender was the only consistent predictor of green consumption across studies, with women displaying more frequent pro-environmental behaviors than men (Testa et al., 2021). Overall, there is no coherent pattern for how socio-demographic variables are associated with environmentally sustainable consumption behaviors across studies (Chekima et al., 2016; Yener et al., 2023).

Within psychological research, the importance of behavior specific determinants of environmentally significant behaviors including, e.g., attitudes, perceived behavioral control, norms, and habits has been emphasized (Verduzco Torres et al., 2022; Rodrigues et al., 2023; Timmer et al., 2023; Brand et al., 2023). The importance of the general antecedents of these behaviors, including values and identity, are furthermore often highly emphasized, since they have the potential to influence a broad set of lifestyle choices via specific behavioral determinants (Bamberg and Möser, 2007; Whitmarsh and O’Neill, 2010; Van der Werff et al., 2013a; Testa et al., 2021). However, as of yet, a framework for psychological determinants of environmental lifestyle behaviors in the domain of physical activity is lacking. Given the importance of identity and motivation for pro-environmental behaviors and participation in physical activity, a framework incorporating these drivers may further the understanding of environmentally significant lifestyle behaviors enabling physical activity.

### 1.3 Theory

How people see themselves, labeled self-identity, has an important influence on behaviors, since people generally want to act in congruence with their self-identity (Stryker and Burke, 2000). Self-identity theory stipulates that people generally have several self-identities (connected to, e.g., the roles they embrace) that varies in importance and salience across situations. The underpinnings of motivations are also key to understanding the roots of behaviors. Self-determination theory (SDT) (Ryan and Deci, 2000) suggests that individual motivations for behaviors originates internally or externally to varying degrees, and a key distinction is made between controlled and autonomous motivation. Controlled motivation refers to responses to external pressures, such as rewards and punishments, or as a way to avoid feelings of guilt when norms prescribe a certain behavior (i.e., external and introjected regulation, respectively). In contrast, autonomous motivation concerns more internalized forms of motivation including inherent aspirations (intrinsic regulation), conformity to who the person is (integrated regulation), or because the behavior is considered important (identified regulation).

A specific form of action-focused self-identity is environmental self-identity, which reflects a general conception of oneself as a person who performs pro-environmental behaviors (Van der Werff et al., 2013a; Vesely et al., 2021). Rooted in biospheric values, a stronger environmental self-identity has been found to be important for a range of pro-environmental and climate friendly behaviors, including travel mode choice and consumption (Gatersleben et al., 2014; Van der Werff et al., 2014; Vesely et al., 2021). However, even with a strong environmental self-identity, the motivation for performing pro-environmental behaviors may still be different across individuals. Given that acting pro-environmentally may be perceived as an obligation and not always enjoyable, environmental self-identity has been found to influence pro-environmental behaviors mainly via an introjected form of motivation (van der Werff et al., 2013a; Floress et al., 2022). However, while Fatoki (2022) confirmed the importance of introjected motivation, intrinsic motivation was also found to be a mediator between environmental self-identity and saving electricity (see also Pelletier et al., 1998).

As the participation in sports and outdoor recreation often requires traveling and materials, the underlying drivers of physical activity may also play a role for environmentally significant behaviors in this domain. Activity specific self-identities, i.e., taking on a role connected to the activity itself such as being an athlete, an outdoor recreationist, or a soccer player, hiker, or climber, has been found to be associated with autonomous motivation and a higher level of involvement in an activity (Rhodes et al., 2020; Lochbaum et al., 2022). While mainly autonomous motivation has been found to be important for exercising, controlled motivation tends to be either unrelated or negatively associated with a higher exercise frequency (Teixeira et al., 2012; Strachan et al., 2013). Nevertheless, it is important to note that different types of motivations may underly sport and outdoor participation (Lynch and Dibben, 2016; Calogiuri and Elliott, 2017).

### 1.4 Aim and hypotheses

The overall aim of the present study is to examine the potential conflicts between a physically active and an environmentally sustainable lifestyle. Since psychological processes may play out differently across domains, e.g., due to differences in salient roles (Whitmarsh et al., 2018; Nielsen et al., 2020), physical activity drivers are expected to be important also for environmentally significant behaviors conducted in this domain. Thus, this study examines how environmental drivers in combination with physical activity drivers, separately and interactively, are associated with environmentally significant lifestyle behaviors associated with physical activity. Specifically, we focus on travel mode choice and consumption of clothes and equipment. In addition, besides investigating the importance of psychological drivers, this study also examines the socio-demographic, physical context, and life situation correlates of these behaviors.

**Figure 1:**
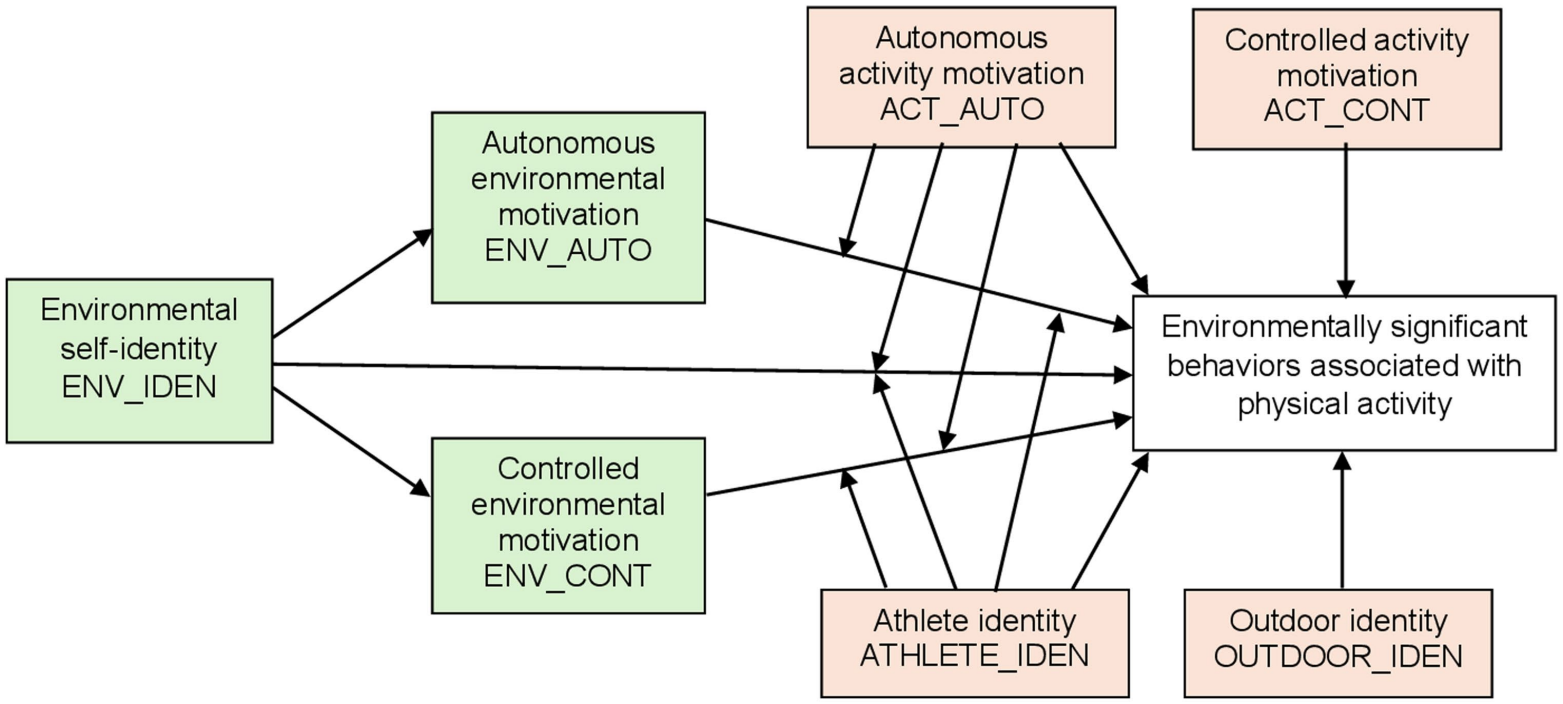
Environmental drivers interacting with physical activity drivers as predictors of environmentally significant behaviors associated with physical activity.

First, we examine the importance of environmental and physical activity drivers for environmentally significant behaviors (Figure 1). Previous research supports the role of environmental self-identity as a driver of pro-environmental behavior both directly, via introjected motivation (i.e., a form of controlled motivation), and through behavioral specific autonomous motivation (Van der Werff et al., 2013a; Fatoki, 2022). We therefore expected environmental self-identity to be associated with autonomous environmental motivation (H1) and with controlled environmental motivation (H2), as well as directly associated with environmentally significant behaviors (H3). In turn, we expected autonomous environmental motivation and environmentally significant behaviors to be associated (H4) and we anticipated a significant relationship between controlled environmental motivation and environmentally significant behaviors (H5).

Autonomous motivation for physical activity, but also activity specific identities, has been found to be important for engaging in physical activity (Teixeira et al., 2012; Lochbaum et al., 2022), while the effect of controlled motivation generally has been insignificant. We expected that physical activity drivers play a role also for the environmentally significant behaviors enabling these activities. More specifically, we expected a direct impact of autonomous activity motivation, controlled activity motivation, athlete identity, and outdoor identity on environmentally significant behaviors (H6, H7, H8, H9, respectively).

We were furthermore interested in examining whether environmental drivers interacted with physical activity drivers in determining environmentally significant behaviors. We therefore tested whether autonomous activity motivation moderated (1) the relationship between environmental self-identity and environmentally significant behaviors (H10), (2) the relationship between autonomous environmental motivation and environmentally significant behaviors (H11), and (3) the relationship between controlled environmental motivation and environmentally significant behaviors (H12), as well as the extent to which athlete identity moderated the same relationships (H13, 14, and 15, respectively). Since higher levels of physical activity may boost environmentally significant behaviors in this domain, and we wanted to isolate the effect of psychological drivers, we examined the stipulated relationships while controlling for hours individuals were involved in physical activity.

Second, we examine the role of socio-demographic, physical contextual, and life situational variables for environmentally significant behaviors, while controlling for hours of physical activity. Several of these variables have been found to be relevant for, e.g., travel mode choice more generally (Susilo et al., 2019; Bi and Romao, 2021), but their impact on trips associated with exercise and outdoor activities, as well as consumption is less clear (Strömblad et al., 2022; Testa et al., 2021). Since organized sport activities have been found to increase unsustainable lifestyle behaviors (Wicker, 2019), we also explored how involvement in outdoor and sport organizations was related to environmentally significant behaviors.

## 2 Methods

### 2.1 Study context

In the present study, we focus on Sweden, which as a case is characterized by both high levels of engagement in physical activities among the general population and at the same time high ambitions to decrease transportation related emissions and to increase the circular use of materials. First, engagement in sport, physical activity, and outdoor recreation is generally relatively widespread in the population - but with a large variation across different groups. The large majority, 80% of the public, report spending time outdoors (e.g., taking walks and trips to the forest) often during holidays, while 50% do this on weekdays (Fredman et al., 2019). Moreover, 75% of the public engage in moderately strenuous physical activity at least once a week, and just over 50% engage in very strenuous activities (The Swedish Sports Confederation, 2021). Second, realizing the political goal for sustainable transportation in Sweden is still distant and many negative environmental impacts needs to be further reduced (e.g., air pollution and emission of greenhouse gases) (Government Bill, 2004: 150; Government Bill, 2008: 93). In the case of exercise and outdoor life travel, car is the dominant travel mode if both traveling by car as driver (27%) and as a passenger (33%) is included (in total 60%), followed by active travel modes, e.g., cycle (27%), and trips made by public transport (10%) (Strömblad et al., 2022). Third, political strivings to facilitate a circular economy focusing on circular flows of material have intensified (European Commission (EC), 2020; Swedish Government Office, 2020). In 2020, two third of Swedish consumers bought at least one, and on average four, used products. Of these, furniture was the dominating product category, followed by electronics, and clothes. This was predominantly an online business, and younger people were overrepresented. However, used clothes constituted only 7% of the clothes market and used sport and leisure products only 4% (Swedish Commerce, 2021).

### 2.2 Procedure and sample

The study was approved by the Swedish Ethical Review Authority (Dnr 2023–01292-01). Following the ethical guidelines as stipulated in the 1964 Declaration of Helsinki, participants were informed about the aim of the study, how personal information was handled, and that participation was voluntary. Before data analyses, the data was pseudonymized to protect the privacy of participants. The web-survey to the general public in Sweden was conducted in the spring of 2023 (between May 12th and July 3rd) by a survey company (Kvalitetsindikator AB). A simple random selection of 8,000 people aged 18–65 was drawn from the Swedish population registry. Initially, respondents were contacted via a mail-out invitation with a link and QR-code to the web-survey. Subsequently, one reminder was sent via regular mail and three through text messages. In total 1,018 respondents filled out the entire survey, resulting in a response rate of approx. 13%.

The socio-demographic profile of the sample is displayed in Table 1. Overall, the sample is fairly representative of the general public, but a larger share lived in large cities and the education level was higher (Statistics Sweden, 2022, 2024).

**Table 1:** Socio-demographic profile of the sample.

Gender (women)	52%
Mean age (years)	48 (13)
University degree	59%
Urban living (50,000 or more inhabitants)	55%
Households with child/children	33%
Membership (own or child) in sport and/or outdoor organization, and/or leader	49%
At least one fossil fuel car in household	71%

### 2.3 Measures

Measures were developed based on previous research and adapted to the current study context and aims. In the questionnaire, physical activity was defined in terms of strenuous activities (e.g., jogging and athletic activities), and moderately strenuous activities (e.g., walking), conducted individually or as part of organized activities. To address the aims of the study, questions covering environmental drivers, physical activity drivers, involvement in physical activity, socio-demographics, the context, life situational variables, and environmentally significant behaviors associated with physical activity were included in the questionnaire. To validate the drivers, questions covering general pro-environmental behaviors and frequency of engaging in athlete and outdoor activities were also included (Supplementary Table S1).

#### 2.3.1 Environmental drivers

Environmental self-identity (ENV_IDEN) was assessed by means of three items (e.g., Acting environmentally-friendly is an important part of who I am) using a five-point response scale (Completely disagree, Completely agree) (*α* = 0.91) (van der Werff et al., 2013a; van der Werff et al., 2013b). Environmental motivation was assessed using six items for autonomous motivation (ENV_AUTO), reflecting internalized motivation (e.g., because it is fun) (*α* = 0.88) and six items for controlled motivation (ENV_CONT), reflecting a higher degree of external regulation (e.g., others say I should) using a five-point scale (Not at all, Completely) (*α* = 0.73) (e.g., Lee et al., 2021; Eriksson et al., 2023).

#### 2.3.2 Physical activity drivers

Two types of self-identity, associated with athletic activities (ATHLETE_IDEN) and outdoor recreation (OUTDOOR_IDEN), respectively, were assessed by means of one item each: “To what extent would you describe yourself in the following ways? As an athlete, As an outdoor person, with answers provided on a five-point scale (Not at all, A little, Partly, A lot, Completely). Physical activity motivation was assessed using 12 comparable items to environmental motivation, reflecting an autonomous motivation (ACT_AUTO) and controlled motivation (ACT_CONT) to be physically active using the same five-point response scale (*α* = 0.93 and *α* = 0.74, respectively).

#### 2.3.3 Environmentally significant behaviors associated with physical activity

Travel mode choice on trips associated with physical activity was measured by means of the question: “How often do you use the below as means of transport on trips to physical activity?” in relation to car use alone, public transportation, and cycle or walk (using a five-point response scale, Very seldom or never, Approx. 1 times/month, Approx. 2–3 times/month, 1–2 times/week, 3–4 times/week, 5 times/week or more). Consumption associated with physical activity was assessed using the following question: “Physical activity requires appropriate clothing and sometimes also other equipment. How often do you do the following?” in relation to buying used material, selling, exchanging or donating used material, and buying new material (using a four-point scale, Never, One or some times/year, Approx. one time/month, Several times/month). Responses were recoded into dummy variables, Very seldom or never = 0 (remaining response options = 1) in relation to travel mode choice, and Never = 0 (remaining response options = 1) in relation to consumption of material.

#### 2.3.4 Socio-demographic, physical context, and life situational variables

Questions about socio-demographics examined in this study included gender, age, and university education. A measure of size of the place the respondents live in (rural versus urban residency, urban = 50,000 inhabitants or more) was used as an indicator of the physical context. Life situation variables included children in the household (dummy), and the respondents’ and/or their children’s membership (and/or leadership position) in sports and/or outdoor organization (dummy).

#### 2.3.5 Other measures

A measure of involvement in physical activity (ACT_HOUR) was created based on the mean of the questions: “How many hours on average in 1 week, do you participate in moderately strenuous activities such as walking?” and “How many hours on average in 1 week, do you participate in very strenuous activities such as jogging?” with answers provided on a four-point scale (0 h, 1–4 h, 5–10 h, 11 h or more) (1–4, *M* = 2.21, *SD* = 0.57). In addition, a general measure of pro-environmental behaviors (PEB) was created by using the following question: “How often do you do the following during a regular week, approximately?” covering seven behaviors in the domains of food consumption, waste reduction, and transportation (*cf.* Whitmarsh et al., 2022). Answers were provided on a five-point scale (Not at all, 1–2 times, 3–4 times, 5–6 times, At least once a day). Two behaviors, car use alone and consumption of red meat, were reversed before all behaviors were summarized (1–35, *M* = 23.39, *SD* = 4.73). The frequency of engaging in athlete and outdoor activities were measured using the question: “How often do you the following?” in relation to six different activities. Answers were provided on a five-point scale (Every day or several times/week, Several times/month, Approx. 1 time/month, One or a few times/year, More seldom or never). Three athlete activities (e.g., exercising individually) (ATHLETE_FREQ) and three outdoor activities (e.g., spend time in forest and land) (OUTDOOR_FREQ) were summarized after the scale had been reversed so that a higher value represents a higher frequency (1–15, *M* = 7.39, *SD* = 2.57 and *M* = 7.40, *SD* = 2.50, respectively).

### 2.4 Analyses

All analyses were conducted using SPSS version 29. Initially, descriptives, including means and standard deviations or frequencies, for all study variables were calculated. Subsequently, the criterion validity of the examined drivers was examined. Associations between the environmental drivers and the general measure of pro-environmental behavior, and associations between the physical activity drivers and physical activity frequency (athlete and outdoor) were assessed by using bivariate correlations (Pearson correlations, *r*).

To examine the environmental and physical activity drivers of environmentally significant behaviors as stated in the first aim, we used PROCESS models (MODEL 17; two mediators and two moderators) with bootstrap (5,000 samples and 95 Confidence Interval, Heteroscedasticity-consistent inference HC4 with centered continuous variables) to estimate six models (Hayes, 2022). This logistic regression path analysis was chosen to enable analyses and significance tests of both mediation and moderation effects. Coefficients (log odds), including standard errors, z values, and confidence intervals are reported in the tables. In addition, significant interactions are plotted across low (one SD below mean), moderate (the mean), and high levels (one SD above the mean) of the examined variables. Predicted probabilities of engaging in environmentally significant behaviors are used to assess the importance of significant interactions as well as for selected main effects.

The models included environmental self-identity as the predictor, and environmentally significant behaviors (car use alone, public transport, cycle/walk, buy new, buy used, and sell used) as dependent variables (binary variables), respectively, whereas autonomous environmental motivation and controlled environmental motivation were included as mediators (M_1_ and M_2_, respectively). Autonomous activity motivation and athlete identity were included as moderators, while controlled activity motivation, outdoor identity and involvement in physical activity were added as covariates. This also allowed us to test the moderated mediation effect, i.e., the conditional indirect effect of moderators on the relationship between environmental self-identity and environmentally significant behavior via environmental motivations (i.e., mediators). Results from a model where outdoor identity (instead of athlete identity) was included as moderator can be found in Supplementary Table S2.

The second aim of the study was examined by means of a binary logistic regression analysis with socio-demographic variables (gender, age, education), physical context variable (place), and life situation variables (child in household, membership in sports and/or outdoor organization) included as independent variables and environmentally significant behaviors as dependent variables, while controlling for involvement in physical activity.

## 3 Results

### 3.1 Descriptives and validation of drivers

Descriptives showed that the autonomous motivation for pro-environmental behaviors was stronger than the controlled motivation (*t*(1012) = 46.26, *p* = 0.001), and that the environmental self-identity measure was close to the center of the scale (Table 2). For physical activity, the autonomous motivation was also stronger than the controlled motivation (*t*(993) = 59.94, *p* = 0.001) and the respondents overall displayed a stronger outdoor identity than an athlete identity (*t*(1012) = 26.03, *p* = 0.001). Around two thirds of the respondents (70%) indicate that they cycle or walk on trips associated with physical activity at least once a month, whereas about half used the car alone. However, less than one third (31%) use public transport on these trips. The majority, 87%, report buying new material once or a couple of times a year, but only 38% buy used material with the same frequency. Moreover, over half (53%) report selling used material (see Table 2).

**Table 2:** Means and standard deviations of drivers, behavioral frequencies, and control variable.

		M (SD)
Environmental drivers	Environmental self-identity ENV_IDEN	3.02 (0.66)
	Environmental autonomous ENV_AUTO	3.67 (0.87)
	Environmental controlled ENV_CONT	2.53 (0.74)
Physical activity drivers	Activity autonomous ACT_AUTO	4.08 (0.95)
	Activity controlled ACT_CONT	2.21 (0.70)
	Athlete identity ATHLETE_IDEN	1.86 (1.12)
	Outdoor identity OUTDOOR_IDEN	2.99 (1.14)

Scale 1–5 (a higher value represents a stronger identity/motivation, respectively).

The significant relationships between the environmental drivers and the general measure of pro-environmental behaviors support their validity as predictors of environmentally significant behaviors (environmental self-identity, *r* = 0.29***, autonomous environmental motivation, *r* = 0.41***, and controlled environmental motivation, *r* = 0.24***). The result for physical activity drivers is, however, more complex, revealing that the physical activity drivers are valid determinants of athlete activities, but that the motivational constructs are less relevant for frequency of engaging in outdoor activities. More specifically, the two forms of motivation are both significantly correlated with frequency of engagement in athlete activities (autonomous activity motivation, *r* = 0.33***, controlled activity motivation, *r* = 0.14***). Autonomous activity motivation does, however, lack a significant correlation with frequency of engaging in outdoor activities (*r* = 0.05, ns) and the correlation between controlled activity motivation and frequency of engaging in outdoor activities is negative and weak (*r* = −0.08*). Nevertheless, athlete identity displays the expected correlation with frequency of engagement in athlete activities (*r* = 0.41***) and outdoor identity displays comparable relationship with frequency of engaging in outdoor activities (*r* = 0.36***). For bivariate correlations between all study variables (see Supplementary Table S3).

### 3.2 Environmental and physical activity drivers

The first aim focused on relationships between drivers and environmentally significant behaviors associated with physical activity. Results revealed a significant relationship between environmental self-identity and autonomous environmental motivation [0.74 (*p* < 0.001, 95% CIs [0.66 0.81], R^2^ = 0.31)] in support of H1 and between environmental self-identity and controlled environmental motivation [0.59 (*p* < 0.001, 95% CIs [0.52 0.66], R^2^ = 0.28] in support of H2. However, the environmental drivers were not associated with all the behaviors (travel mode choice, Table 3, consumption, Table 4). Specifically, while we found no support for a direct impact of environmental self-identity on the behaviors in any of the models (H3), autonomous environmental motivation was found to be negatively associated with using the car alone (in support of H4) and controlled environmental motivation was positively associated with buying and selling used material (in support of H5).

**Table 3:** The impact of environmental drivers and physical activity drivers on travel behavior associated with physical activity, while controlling for hours of physical activity (autonomous activity motivation and athlete identity as moderators).

	Car use alone			Public transport			Cycle/walk		
	Coeff	Z value	CI 95%	Coeff	Z value	CI 95%	Coeff	Z value	CI 95%
Constant	−0.89 (0.47)	−1.90	−1.81 0.03	−2.43 (0.50)	−4.90***	−3.41 -1.46	−0.08 (0.51)	−0.17	−1.08 0.91
H3 ENV_IDEN	−0.25 (0.13)	−1.84	−0.51 0.02	0.10 (0.14)	0.74	−0.17 0.38	0.17 (0.15)	1.20	−0.11 0.46
H4 ENV_AUTO	−0.27 (0.11)	−2.42*	−0.48 -0.05	0.07 (0.12)	0.59	−0.16 0.30	−0.16 (0.12)	−1.30	−0.39 0.08
H5 ENV_CONT	0.05 (0.13)	0.39	−0.21 0.31	0.14 (0.14)	0.97	−0.14 0.41	0.20 (0.15)	1.37	−0.09 0.48
H6 ACT_AUTO	−0.02 (0.10)	−0.21	−0.21 0.17	−0.14 (0.10)	−1.36	−0.35 0.06	0.33 (0.10)	3.28***	0.13 0.52
H7 ACT_CONT	0.30 (0.12)	2.44*	0.06 0.54	0.58 (0.13)	4.51***	0.33 0.83	0.03 (0.13)	0.19	−0.24 0.29
H8 ATHLETE_IDEN	0.38 (0.07)	5.30***	0.24 0.53	0.11 (0.07)	1.48	−0.04 0.26	0.15 (0.08)	1.79	−0.01 0.31
H9 OUTDOOR_IDEN	0.18 (0.07)	2.69**	0.05 0.31	−0.11 (0.07)	−1.49	−0.24 0.03	0.00 (0.07)	−0.02	−0.14 0.14
H10 ENV_IDEN*ACT_AUTO	−0.45 (0.15)	−3.00**	−0.74 -0.16	0.00 (0.15)	0.02	−0.30 0.31	0.07 (0.15)	0.47	−0.22 0.36
H11 ENV_CONT*ACT_AUTO	0.04 (0.13)	0.32	−0.22 0.30	−0.08 (0.14)	−0.58	−0.36 0.20	0.04 (0.14)	0.30	−0.23 0.31
H12 ENV_AUTO*ACT_AUTO	0.12 (0.11)	1.10	−0.10 0.35	0.17 (0.12)	1.41	−0.07 0.40	−0.10 (0.11)	−0.84	−0.32 0.13
H13 ENV_IDEN*ATHLETE_IDEN	0.03 (0.13)	0.20	−0.22 0.27	0.06 (0.12)	0.51	−0.18 0.30	−0.09 (0.14)	−0.63	−0.36 0.18
H14 ENV_CONT*ATHLETE_IDEN	0.14 (0.10)	1.21	−0.09 0.38	0.10 (0.12)	0.84	−0.13 0.33	−0.16 (0.13)	−1.26	−0.41 0.09
H15 ENV_AUTO*ATHLETE_IDEN	0.00 (0.10)	0.03	−0.20 0.20	−0.04 (0.10)	−0.37	−0.24 0.16	0.07 (0.11)	0.65	−0.15 0.29
ACT_HOUR	−0.14 (0.14)	−1.01	−0.42 0.14	0.25 (0.15)	1.65	−0.05 0.55	0.44 (0.16)	2.76**	0.13 0.75
Pseudo R^2^_N_	0.12			0.09			0.10		
-2LL	1288.00			1156.26			1141.53		

* *p* < 0.05, **, *p* < 0.01, *** *p* < 0.001. Environmental self-identity (ENV_IDEN), Environmental autonomous motivation (ENV_AUTO), Environmental controlled motivation (ENV_CONT), Activity autonomous motivation (ACT_AUTO), Activity controlled motivation (ACT_CONT), Athlete identity (ATHLETE_IDEN), Outdoor identity (OUTDOOR_IDEN), Physical activity involvement (ACT_HOUR).

**Table 4:** The impact of environmental drivers and physical activity drivers on consumption behaviors associated with physical activity, while controlling for hours of physical activity (autonomous activity motivation and athlete identity as moderators).

	Buy new			Buy used			Sell used		
	Coeff	Z value	CI 95%	Coeff	Z value	CI 95%	Coeff	Z value	CI 95%
Constant	0.44 (0.75)	0.59	−1.02 1.91	−2.85 (0.50)	−5.74***	−3.82 -1.88	−1.05 (0.47)	−2.27*	−1.97 -0.14
H3 ENV_IDEN	0.14 (0.24)	0.56	−0.34 0.61	0.13 (0.14)	0.90	−0.15 0.40	0.16 (0.13)	1.23	−0.10 0.43
H4 ENV_AUTO	−0.19 (0.20)	−0.93	−0.59 0.21	0.03 (0.12)	0.24	−0.20 0.26	0.04 (0.11)	0.36	−0.17 0.25
H5 ENV_CONT	0.00 (0.24)	0.00	−0.47 0.47	0.49 (0.14)	3.51***	0.22 0.77	0.27 (0.13)	2.06*	0.01 0.53
H6 ACT_AUTO	0.61 (0.13)	4.54***	0.34 0.87	0.01 (0.11)	0.09	−0.20 0.22	0.15 (0.10)	1.58	−0.04 0.34
H7 ACT_CONT	0.33 (0.20)	1.66	−0.06 0.72	0.28 (0.13)	2.23*	0.03 0.53	0.10 (0.12)	0.79	−0.14 0.34
H8 ATHLETE_IDEN	0.38 (0.15)	2.51*	0.08 0.69	0.04 (0.07)	0.54	−0.10 0.18	0.16 (0.07)	2.26*	0.02 0.30
H9 OUTDOOR_IDEN	0.03 (0.10)	0.33	−0.16 0.23	0.36 (0.07)	5.14***	0.23 0.50	0.21 (0.07)	3.15**	0.08 0.34
H10 ENV_IDEN*ACT_AUTO	−0.29 (0.19)	−1.54	−0.66 0.08	−0.36 (0.16)	−2.17*	−0.68 -0.04	−0.25 (0.15)	−1.67	−0.55 0.04
H11 ENV_CONT*ACT_AUTO	−0.06 (0.18)	−0.32	−0.42 0.30	−0.04 (0.14)	−0.26	−0.32 0.25	−0.07 (0.14)	−0.54	−0.34 0.19
H12 ENV_AUTO*ACT_AUTO	0.06 (0.15)	0.40	−0.23 0.36	0.18 (0.12)	1.45	−0.06 0.42	0.08 (0.11)	0.70	−0.14 0.30
H13 ENV_IDEN*ATHLETE_IDEN	0.28 (0.25)	1.12	−0.21 0.77	0.17 (0.12)	1.45	−0.06 0.41	−0.04 (0.12)	−0.33	−0.28 0.20
H14 ENV_CONT*ATHLETE_IDEN	−0.26 (0.24)	−1.06	−0.74 0.22	−0.40 (0.11)	−3.48***	−0.62 -0.17	−0.04 (0.11)	−0.31	−0.26 0.19
H15 ENV_AUTO*ATHLETE_IDEN	−0.08 (0.21)	−0.36	−0.49 0.34	0.15 (0.10)	1.53	−0.04 0.35	0.18 (0.10)	1.85	−0.01 0.38
ACT_HOUR	0.46 (0.23)	1.98*	0.00 0.92	0.25 (0.15)	1.67	−0.04 0.54	0.16 (0.14)	1.12	−0.12 0.44
Pseudo R^2^_N_	0.23			0.16			0.12		
-2LL	649.66			1193.31			1280.10		

* *p* < 0.05, **, *p* < 0.01, *** *p* < 0.001. Environmental self-identity (ENV_IDEN), Environmental autonomous motivation (ENV_AUTO), Environmental controlled motivation (ENV_CONT), Activity autonomous motivation (ACT_AUTO), Activity controlled motivation (ACT_CONT), Athlete identity (ATHLETE_IDEN), Outdoor identity (OUTDOOR_IDEN), Physical activity involvement (ACT_HOUR).

Several of the physical activity drivers were associated with the environmentally significant behaviors. We found that autonomous activity motivation was associated with a higher likelihood of cycling/walking and buying new material (both in support of H6). At average levels of the other determinants in the model, those with a strong autonomous activity motivation (one SD above the mean) were 13% more likely to cycle/walk and 10% more likely to buy new material compared to those with a weak autonomous motivation (one SD below the mean). In contrast, controlled activity motivation was associated with a higher likelihood of using the car alone, using public transport, and buying used material (in support of H7). In addition, athlete identity was associated with a higher likelihood of using the car alone, buying new material, and selling used material (in support of H8). Outdoor identity was associated with a higher likelihood of using the car alone and selling used material, but also buying used, rather than new, material (in support of H9).

**Figure 2:**
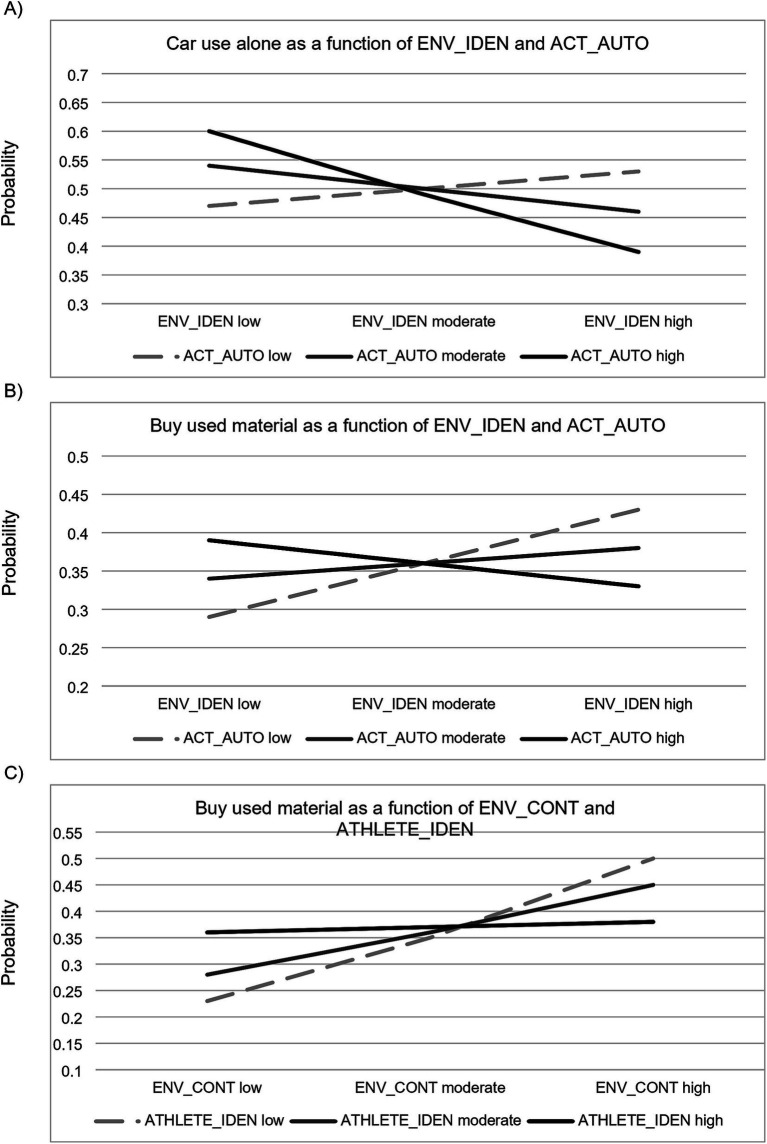
Illustration of an environmental driver increasing the probability for acting pro-environmentally more if the motivation for physical activity is strong: The probability of using the car alone as a function of environmental self-identity (ENV_IDEN) and autonomous activity motivation (ACT_AUTO) **(A)**, The probability of buying used material as a function of controlled environmental motivation (ENV_CONT) and athlete identity (ATHLETE_IDEN). Illustration of an environmental driver increasing the probability for acting pro-environmentally more if the motivation for physical activity is weak **(B)**, The probability of buying used material as a function of environmental self-identity (ENV_IDEN) and autonomous activity motivation (ACT_AUTO) **(C)**.

**Figure 3:**
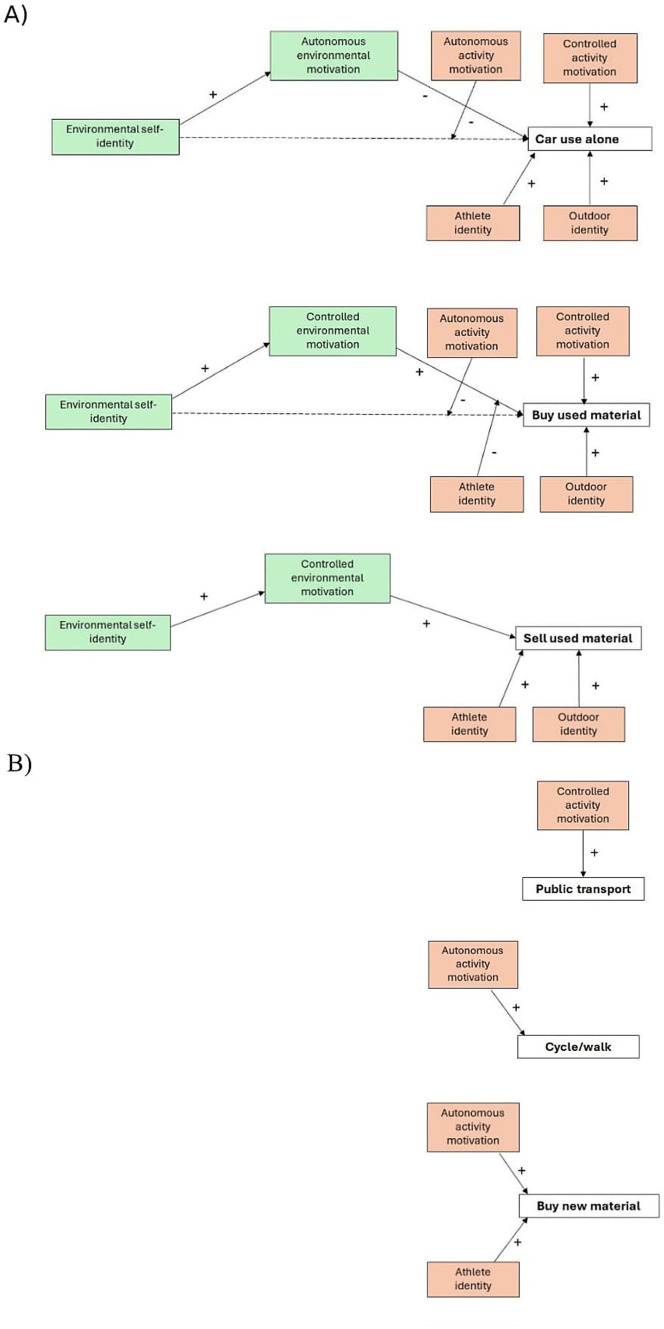
Schematic overview of results for integrated models in relation to six environmentally significant behaviors associated with physical activity. Models where environmental and physical activity drivers are important for environmentally significant behaviors **(A)** and Models where only physical activity drivers are important for environmentally significant behaviors **(B)**.

The results also revealed a few significant interactions (Figure 2). First, the interaction between environmental self-identity and autonomous activity motivation was significant in relation to using the car alone. This suggests that environmental self-identity reduces the likelihood of using the car particularly among those with a stronger autonomous motivation for physical activity (supporting H10 in relation to car use alone) (Figure 2A). The moderated mediation effects were, however, not significant. Among respondents with a strong autonomous motivation, those with a strong environmental self-identity were 21% less likely to use the car alone compared to those with a weak environmental self-identity. Second, in relation to buying used material, the interaction between environmental self-identity and autonomous activity motivation was significant. This suggests that a stronger environmental self-identity increases the likelihood of buying used material more among those with a weak autonomous motivation for physical activity (supporting H10 in relation to buying used material) (Figure 2B). Again, the moderated mediation effects were not significant. The interaction suggests that among respondents with a weak autonomous activity motivation, those with a strong environmental self-identity were 14% more likely to buy used material than those with a weak environmental self-identity. Third, also the interaction between controlled environmental motivation and athlete identity was significant, suggesting that a stronger controlled environmental motivation increases the likelihood of buying used material more among those with a weak athlete identity (supporting H14 in relation to buy used material). This time, the moderated mediation index was significant, revealing that the effect of environmental self-identity on buying used products via controlled environmental motivation is moderated by athlete identity (−0.18 95% CI −0.30 −0.08) (Figure 2C). Among respondents with a weak athlete identity, those with a strong controlled environmental motivation were 27% more likely to buy used material than those with a weak motivation. A table providing an overview of support for hypotheses in relation to the different behaviors can be found in Supplementary Table S4 and a schematic overview of results for the integrated models where both environmental and physical activity drivers are important (panel A) and models where only physical activity drivers play a role (panel B) is displayed in Figure 3.

### 3.3 The role of socio-demographic, physical, and life situation variables

Analyses of the second aim revealed that after controlling for hours of physical activity, women were less likely to use the car alone, while respondents with children in the household were more likely to use the car alone (Table 5). Moreover, gender and having children in the household were also associated with buying and selling used material. Younger respondents were more likely to use public transport and cycle/walk, as well as to buy used material. Respondents with a university education were less likely to use the car. Moreover, being an urban resident was associated with being more likely to use public transport and cycle/walk, as well as being less likely to use the car. Nevertheless, urban respondents were also more likely to buy new material. Members of sport or outdoor organization were more likely to use the car alone and buy new material, but also to cycle/walk as well as to buy and sell used material. Hence, even after controlling for hours of physical activity, membership displayed significant positive relationships with all behaviors (sustainable and unsustainable) except the use of public transport.

**Table 5:** Socio-demographic, physical context, and life situation variables as predictors of environmentally significant behaviors associated with physical activity, while controlling for hours of physical activity.

	Car use alone			Public transport			Cycle/walk		
	B (SE)	Wald	Exp (B)	B (SE)	Wald	Exp (B)	B (SE)	Wald	Exp (B)
Constant	−0.44 (0.44)	0.99	0.64	−0.50 (0.47)	1.15	0.61	−0.37 (0.49)	0.57	0.69
Gender	−0.41 (0.14)	8.89**	0.67	0.21 (0.15)	1.98	1.23	0.09 (0.15)	0.37	1.10
Age	0.00 (0.01)	0.22	1.00	−0.03 (0.01)	26.21***	0.97	−0.01 (0.01)	5.53*	0.99
University education	−0.35 (0.15)	5.77*	0.71	0.22 (0.16)	2.00	1.25	−0.30 (0.16)	3.76	0.74
Urban	−0.54 (0.14)	15.34***	0.58	0.71 (0.15)	21.76***	2.03	0.67 (0.15)	21.00***	1.96
Child in household	0.40 (0.15)	6.87**	1.49	−0.10 (0.16)	0.37	0.91	−0.05 (0.17)	0.08	0.95
Member	0.81 (0.14)	32.14***	2.24	−0.12 (0.15)	0.59	0.89	0.39 (0.16)	6.24*	1.48
ACT_HOUR	0.22 (0.13)	3.05	1.25	0.19 (0.14)	2.05	1.22	0.69 (0.15)	22.83***	2.00
Pseudo R^2^_N_	0.12***			0.10***			0.10***		
-2LL	1277.93			1139.05			1134.33		

	Buy new			Buy used			Sell used		
	B (SE)	Wald	Exp (B)	B (SE)	Wald	Exp (B)	B (SE)	Wald	Exp (B)
Constant	−0.66 (0.66)	0.98	0.52	−0.44 (0.45)	0.95	0.64	−1.53 (0.45)	11.86	0.22
Gender	−0.04 (0.20)	0.03	0.97	0.60 (0.14)	17.68***	1.83	0.71 (0.14)	26.57***	2.03
Age	−0.01 (0.01)	1.45	0.99	−0.03 (0.01)	32.58***	0.97	0.00 (0.01)	0.32	1.00
University education	0.23 (0.21)	1.21	1.26	−0.23 (0.15)	2.32	0.80	−0.16 (0.14)	1.26	0.85
Urban	0.50 (0.20)	6.12*	1.65	−0.22 (0.14)	2.25	0.81	−0.09 (0.14)	0.42	0.92
Child in household	0.36 (0.26)	1.97	1.43	0.57 (0.16)	13.54***	1.77	0.63 (0.15)	16.50***	1.87
Member	1.14 (0.24)	23.16***	3.13	0.42 (0.15)	7.83**	1.52	0.44 (0.14)	9.63**	1.55
ACT_HOUR	1.01 (0.21)	22.99***	2.75	0.43 (0.13)	10.37***	1.53	0.52 (0.13)	16.40***	1.68
Pseudo R^2^_N_	0.17***			0.14***			0.10***		
-2LL	676.87			1203.89			1285.04		

* *p* < 0.05, ** *p* < 0.01, *** *p* < 0.001. Physical activity involvement (ACT_HOUR).

## 4 Discussion

Good health among people for which an active lifestyle is important, should not come at the cost of environmental goals. The global sustainability goals emphasize the importance of good health among people (SDG 3), but also the need for responsible consumption (SDG 12), mitigation of climate change (SDG 13) and protection of water and land (SDG 14 and 15) (United Nations, 2015). In this study, we examined the potential conflicts between these strivings on an individual level. Results revealed that underlying motives for physical activity may contribute to an unsustainable lifestyle, but also that there are exceptions, and that this impact in some instances may be alleviated by the specific drivers underlying a pro-environmental lifestyle.

In this study, the environmental drivers were found to be associated with only a few of the examined environmentally significant behaviors in the domain of physical activity. As neither the use of public transport, the choice of using active transportation modes, nor the purchase of new material, were related to the environmental drivers, this indicates that environmental reasoning may be less important for environmental lifestyle behaviors in this domain. Results further showed that the physical activity drivers were relevant for the examined behaviors, even after controlling for involvement in physical activity. Autonomous motivation for physical activity was, for example, associated with a higher likelihood of using active travel modes, serving as an example of when a driver of physical activity also can promote environmental sustainability. Nevertheless, autonomous activity motivation was also associated with a higher likelihood of buying new materials (representing a linear rather than a circular consumption pattern) which illustrates a potential incompatibility between drivers of physical activity and environmental sustainability. Since there are psychological benefits associated with autonomous motivation (e.g., subjective well-being and relatedness within a social group) (Ryan and Deci, 2000), there is as such a need to consider how to disconnect the importance of this motivation from unsustainable behaviors, without reducing autonomous motivation for physical activity. The results furthermore revealed that the activity identities facilitated both some of the more sustainable behaviors (e.g., selling used materials) and some of the unsustainable behaviors (most notably using the car). Athlete identity was also associated with a higher likelihood of buying new material. To more fully understand the compatibility between activity identities and environmental sustainability, it may, for example, be important to study how diverse consumption logics and notions of stewardship are integrated into different identities (Wang et al., 2023).

The results further revealed significant interaction effects between environmental and physical activity drivers in relation to environmentally significant behaviors in this domain. For example, a strong environmental self-identity decreased the likelihood of using the car for physical activity especially among those with a stronger autonomous activity motivation. Hence, results suggest that not only can a stronger environmental autonomous motivation dampen car use, but also that a strong environmental self-identity may contribute to this aim among those with an internalized motivation to engage in physical activity. Nevertheless, a stronger environmental self-identity mainly increased the probability of buying used material among those with a lower level of internalized motivation for physical activity. Given that environmental drivers lessen the negative impact of physical activity drivers, facilitating environmental drivers is important also in this domain. Interdisciplinary integration may aid to further examine how motivations underlying behaviors in a specific domain may interfere with more general environmental drivers (Nielsen et al., 2020).

As there is an urgent need for sustainability transitions both in relation to travel behavior and consumption, research investigating the decision-making underlying travel mode choice (e.g., Bamberg and Schmidt, 2003; Eriksson and Forward, 2011; Klöckner, 2014; Wallén Warner et al., 2021) and the determinants of linear versus circular consumption patterns (e.g., Arman and Mark-Herbert, 2022; Brand et al., 2023; Fors et al., 2023) is critical. While previous studies have confirmed that socio-demographic, contextual, and life situation constraints are associated with travel mode choice, the present study also revealed that consumption is associated with socio-demographics and the life situation. Being younger was associated with buying used material and being a woman and having children in the household was associated with both buying and selling used material. In contrast, urban residency was associated with buying new material. This result may partly be due to restrictions reflected by these variables (e.g., lower income or lack of access to a service). Being a member in a sport and/or outdoor organization was also found to be associated with almost all environmental behaviors (even after controlling for hours of physical activity), with particularly strong relations with car use alone and buying new material. Membership therefore seems to facilitate various behaviors enabling these activities independent of their environmental impact. More knowledge on how organizational variables (e.g., sustainability ambitions and strategies) within these organizations are associated with the environmentally significant behaviors of individual members may provide a basis to further clarify this pattern of results.

There are limitations to consider when interpreting the results from this study. The response rate was low, potentially because this was an online study only, and the questionnaire may have been perceived to be too long. Although the sample was reasonably representative of the population in relation to socio-demographic characteristics, it was slightly more educated and more urban, which might be associated with more pro-environmental travel modes in the sample compared to the general population. One may also expect that people interested in either environmental sustainability, sports, and outdoor recreation, were more likely to participate. Nevertheless, results regarding the reliance on active travel modes and the car, and higher involvement in linear than circular consumption are in line with expectations. Moreover, the present study is mostly focusing on how different psychological and other variables are associated with environmentally significant behaviors, suggesting that slight deviations only will have minor impacts on interpretations. Furthermore, even though the tested models are theoretically derived, data is correlational and cannot support interpretations regarding causality. Given the relatively low explained variance in some of the models, future research also needs to study additional mediators between environmental drivers and environmentally significant behaviors associated with physical activity, considering, e.g., behavioral specific motives (Fallah Zavareh et al., 2020).

Whereas, for example, issues of equality have been a concern in the sports sector for a long time, the importance of environmental sustainability is more recent (Larneby et al., 2022). As this study found membership in sport and outdoor organizations to be associated with environmentally significant behaviors in this domain (positively in some cases but negatively in others), these organizations may potentially be used as platforms to further environmental sustainability. Given that sport organizations in Sweden engage almost a third of the population (3 million) (The Swedish Sports Confederation, 2024) and that only a single umbrella organization for outdoor life in Sweden engage 1.8 million people (The Swedish Outdoor Association, 2024), this influence could potentially be considerable. This work may, for example, be facilitated by drawing on the COM-B (Capability, Opportunity, Motivation, and Behavior) framework (Michie et al., 2011), which outlines the importance of facilitating opportunities (e.g., ensuring that the context and social norms support pro-environmental behaviors), capabilities (e.g., through increased knowledge and efficacy beliefs), and motivations (both conscious and automatic) to encourage behaviors. As part of the motivational basis of behaviors, the present study furthermore supports the need for organizations to highlight their past pro-environmental behaviors, which might strengthen the environmental self-identity among members and potentially increase the frequency of pro-environmental behaviors (van der Werff et al., 2014). Working strategically and operationally on environmental issues within organizations may also contribute to the inclusion of environmental concerns as part of activity identities (*cf.* Samuel et al., 2022). If members have strong feelings of belonging to the organization it is also possible to not only exert external pressure to increase pro-environmental behavior (through, e.g., pro-environmental social norms), but also to facilitate the internalization of environmental motivations through autonomy supporting strategies (Ryan and Deci, 2000).

## Data Availability

The raw data supporting the conclusions of this article will be made available by the authors, without undue reservation.

## References

[ref1] Arman S. M. Mark-Herbert C. (2022). Ethical pro-environmental self-identity practice: the case of second-hand products. Sustain. For. 14:2154. doi: 10.3390/su14042154

[ref2] Backman E. Svensson D. (2022). Where does environmental sustainability fit in the changing landscapes of outdoor sports? An analysis of logics of practice in artificial sport landscapes. Sport Educ. Soc. 28, 727–740. doi: 10.1080/13573322.2022.2073586

[ref3] Bamberg S. Möser G. (2007). Twenty years after Hines, Hungerford, and Tomera: a new meta-analysis of psycho-social determinants of pro-environmental behaviour. J. Environ. Psychol. 27, 14–25. doi: 10.1016/j.jenvp.2006.12.002

[ref4] Bamberg S. Schmidt P. (2003). Incentives, morality, or habit? Predicting students’ Car use for university routes with the models of Ajzen, Schwartz, and Triandis. Environ. Behav. 35, 264–285. doi: 10.1177/0013916502250134

[ref5] Beery T. Wolf-Watz D. (2014). Nature to place: rethinking the environmental connectedness perspective. J. Environ. Psychol. 40, 198–205. doi: 10.1016/j.jenvp.2014.06.006

[ref6] Bernard P. Chevance G. Kingsbury C. Gadais T. Dancause K. Villarino R.. (2022). Climate change: the next game changer for sport and exercise psychology. Ger. J. Exerc. Sport Res. 54, 6–11. doi: 10.1007/s12662-022-00819-w PMC9098786 40479168

[ref7] Bi Y. Romao J. (2021). Soft is better: determinants of preferences for non-motorized forms of transportation in urban tourism destinations. Sustain. For. 13:11944. doi: 10.3390/su132111944

[ref8] Brand S. Jacobs B. Taljaard-Swart H. (2023). I rent, swap or buy second-hand – comparing antecedents for online collaborative clothing consumption models. Int. J. Fash. Des. Technol. Educ. 16, 275–287. doi: 10.1080/17543266.2023.2180541

[ref9] Calogiuri G. Elliott L. R. (2017). Why do people exercise in natural environments? Norwegian adults’ motives for nature-, gym-, and sports-based exercise. Int. J. Environ. Res. Public Health 14:377. doi: 10.3390/ijerph14040377, PMID: 28375192 PMC5409578

[ref10] Charreire H. Roda C. Feuillet T. Piombini A. Bardos H. Rutter H.. (2021). Walking, cycling, and public transport for commuting and non-commuting travels across 5 European urban regions: modal choice correlates and motivations. J. Transp. Geogr. 96:103196. doi: 10.1016/j.jtrangeo.2021.103196

[ref11] Chekima B. Wafa S. A. Aisat Igau O. Chekima S. Sondoh S. L. Jr. (2016). Examining green consumerism motivational drivers: does premium price and demographics matter to green purchasing? J. Clean. Prod. 112, 3436–3450. doi: 10.1016/j.jclepro.2015.09.102

[ref13] Cunningham G. McCullough B. P. Hohensee S. (2020). Physical activity and climate change attitudes. Clim. Chang. 159, 61–74. doi: 10.1007/s10584-019-02635-y

[ref14] Engström L.-M. Redelius K. Larsson H. (2018). Logics of practice in movement culture: Lars-Magnus Engström’s contribution to understanding participation in movement cultures. Sport Educ. Soc. 23, 892–904. doi: 10.1080/13573322.2017.1290597

[ref15] Eriksson L. Forward S. (2011). Is the intention to travel in a pro-environmental manner and the intention to use the car determined by different factors? Trans. Res. D Trans. Environ. 16, 372–376. doi: 10.1016/j.trd.2011.02.003

[ref16] Eriksson L. Månsson J. Liljebäck N. Sandström C. Johansson M. Eklund A.. (2023). How to involve hunters in the adaptive flyway management of geese: the importance of structural, situational, and psychological factors. Sci. Rep. 13:7112. doi: 10.1038/s41598-023-33846-0, PMID: 37130869 PMC10154402

[ref17] European Commission (EC) (2020). A new circular economy action plan for a cleaner and more competitive Europe. Brussels: European Commission.

[ref18] Fallah Zavareh M. Mehdizadeh M. Nordfjærn T. (2020). Active travel as a pro-environmental behaviour: an integrated framework. Transp. Res. Part D: Transp. Environ. 84:102356. doi: 10.1016/j.trd.2020.102356

[ref19] Fatoki O. (2022). Environmental self-identity and energy saving behaviour of hotel emplyees: the mediating role of intrinsic motivation. Geo J. Tour. Geosites 42, 743–750. doi: 10.30892/gtg.422spl13-884

[ref20] Floress K. Shwom R. Caggiano H. Slattery J. Cuite C. Schelly C.. (2022). Habitual food, energy, and water consumption behaviors among adults in the United States: comparing models of values, norms, and identity. Energy Res. Soc. Sci. 85:102396. doi: 10.1016/j.erss.2021.102396

[ref21] Fors P. Nuur A. Randia F. (2023). Conceptualising the peer-to-peer second-hand practice-as-entity. Clean. Respons. Consum. 9:100119. doi: 10.1016/j.clrc.2023.100119

[ref22] Fredman P. Ankre R. Chekalina T. (2019). *Friluftsliv 2018. Nationell undersökning av svenska folkets friluftsvanor.* [outdoor life 2018. A national investigation of outdoor habits among Swedish people.] rapport 6887. Stockholm: Naturvårdsverket.

[ref24] Gatersleben B. Murtagh N. Abrahamse W. (2014). Values, identity and pro-environmental behaviour. Contemp. Soc. Sci. 9, 374–392. doi: 10.1080/21582041.2012.682086

[ref25] Government Bill (2004). 150. Svenska miljömål – ett gemensamt uppdrag. Stockholm: Regeringen.

[ref26] Government Bill (2008). Mål för framtidens resor och transporter. Stockholm: Regeringen.

[ref28] Hayes A. F. (2022). Introduction to mediation, moderation, and conditional process analysis: A regression-based approach. New York: The Guilford Press.

[ref29] Høyem J. (2020). Outdoor recreation and environmentally responsible behavior. J. Outdoor Recreat. Tour. 31:100317. doi: 10.1016/j.jort.2020.100317

[ref30] Hutchinson J. Prady S. Smith M. A. White P. C. L. Graham H. M. (2015). A scoping review of observational studies examining relationships between environmental behaviors and health behaviors. Int. J. Environ. Res. Public Health 12, 4833–4858. doi: 10.3390/ijerph120504833, PMID: 25950651 PMC4454941

[ref31] Juschten M. Preyer B. (2023). Exploring constraints and coping strategies of outdoor recreation trips accessible by public transport: a walk-along and ride-along study in Austria. J. Outdoor Recreat. Tour. 43:100669. doi: 10.1016/j.jort.2023.100669

[ref32] Kawgan-Kagan I. (2020). Are women greener than men? A preference analysis of women and men from major German cities over sustainable urban mobility. Transp. Res. Interdiscip. Perspect. 8:100236. doi: 10.1016/j.trip.2020.100236

[ref34] Klöckner C. A. (2014). The dynamics of purchasing an electric vehicle – a prospective longitudinal study of the decision-making process. Trans. Res. F Traffic Psychol. Behav. 24, 103–116. doi: 10.1016/j.trf.2014.04.015

[ref35] Larneby M. Radmann J. Hedenborg S. (2022). Startskottet har gått. Specialidrottsförbundens arbete med ekologisk hållbarhet [Starting signal. Special sports associations work on ecological sustainability]. Mistra Sport Outdoors Rapport 2022:1.

[ref36] Larson L. R. Stedman R. C. Cooper C. B. Decker D. J. (2015). Understanding the multi-dimensional structure of pro-environmental behavior. J. Environ. Psychol. 43, 112–124. doi: 10.1016/j.jenvp.2015.06.004

[ref37] Larson L. R. Whiting J. W. Green G. T. (2011). Exploring the influence of outdoor recreation participation on pro-environmental behaviour in a demographically diverse population. Local Environ. 16, 67–86. doi: 10.1080/13549839.2010.548373

[ref39] Lee C.-K. Olya H. Ahmad M. S. Kim K. H. Oh M. J. (2021). Sustainable intelligence, destination social responsibility, and pro-environmental behaviour of visitors: evidence from an eco-tourism site. J. Hosp. Tour. Manag. 47, 365–376. doi: 10.1016/j.jhtm.2021.04.010

[ref40] Lochbaum M. Cooper S. Limp S. (2022). The athletic identity measurement scale: a systematic review with Meta-analysis from 1993 to 2021. Eur. J. Investig. Health Psychol. Educ. 12, 1391–1414. doi: 10.3390/ejihpe12090097, PMID: 36135235 PMC9497853

[ref41] Lynch P. Dibben M. (2016). Exploring motivations for adventure recreation events: a New Zealand study. Ann. Leis. Res. 19, 80–97. doi: 10.1080/11745398.2015.1031804

[ref42] Michie S. van Stralen M. M. West R. (2011). The behaviour change wheel: a new method for characterising and designing behaviour change interventions. Implement. Sci. 6:42. doi: 10.1186/1748-5908-6-42 21513547 PMC3096582

[ref43] Nielsen K. S. Clayton S. Stern P. C. Dietz T. Capstick S. Whitmarsh L. (2020). How psychology can help limit climate change. Am. Psychol. 76, 130–144. doi: 10.1037/amp0000624 32202817

[ref44] Pelletier L. G. Tuson K. M. Oels I. R.-D. K. Beaton A. M. (1998). Why are you doing things for the environment? The motivation toward the environment scale (MTES). J. Appl. Soc. Psychol. 28, 437–468. doi: 10.1111/j.1559-1816.1998.tb01714.x

[ref46] Renninger D. Kelso A. Reimers A. K. Marzi I. Beck F. Engels E. S. (2022). Motivation and active travel in adolescent girls and boys in Germany – findings from the ARRIVE study. Trans. Res. F Traffic Psychol. Behav. 90, 425–437. doi: 10.1016/j.trf.2022.09.015

[ref47] Rhodes R. E. Liu S. Lithopoulos A. Garcia-Barrera M. A. (2020). Correlates of perceived physical activity transitions during the COVID-19 pandemic among Canadian adults. Appl. Psychol. Hlth We 12, 1157–1182. doi: 10.1111/aphw.12236, PMID: 33006279 PMC7537295

[ref50] Rodrigues M. Proença J. F. Macedo R. (2023). Determinants of the purchase of secondhand products: an approach by the theory of planned behaviour. Sustain. For. 15:10912. doi: 10.3390/su151410912

[ref51] Ryan R. M. Deci E. L. (2000). Self-determination theory and the facilitation of intrinsic motivation, social development, and well-being. Am. Psychol. 55, 68–78. doi: 10.1037//0003-066x.55.1.68, PMID: 11392867

[ref52] Samuel A. McGouran C. Thomas R. J. Reginald G. White T. (2022). “The club on the hill”: footballing place as an arena for sustainable and ethical action. Qual. Mark. Res. 25, 570–584. doi: 10.1108/QMR-01-2022-0015

[ref53] Sandberg M. (2021). Sufficiency transitions: a review of consumption changes for environmental sustainability. J. Clean. Prod. 293:126097. doi: 10.1016/j.jclepro.2021.126097

[ref54] Statistics Sweden (2022). Statistiska tätorter och småorter 2020. [localities and urban areas 2020.] MI 38 2020A02. Örebro: SCB.

[ref55] Statistics Sweden (2024). Utbildningsnivån i Sverige. [Educational level in Sweden.]. Available at: https://www.scb.se/hitta-statistik/sverige-i-siffror/utbildning-jobb-och-pengar/utbildningsnivan-i-sverige/ (Accessed January 26, 2024).

[ref56] Stern P. C. (2000). Toward a coherent theory of environmentally significant behavior. J. Soc. Issues 56, 407–424. doi: 10.1111/0022-4537.00175

[ref57] Strachan S. M. Fortier M. S. Perras M. G. M. Lugg C. (2013). Understanding variations in exercise-identity strength through identity theory and self-determination theory. Int. J. Sport Exerc. Psychol. 11, 273–285. doi: 10.1080/1612197X.2013.749005

[ref58] Strömblad E. Winslott Hiserlius L. Smidfelt Rosqvist L. Svensson H. (2022). Characteristics of everyday leisure trips by Car in Sweden – implications for sustainability measures. Promet Zagreb 34, 657–672. doi: 10.7307/PTT.V34I4.4039

[ref59] Stryker S. Burke P. J. (2000). The past, present, and future of an identity theory. Soc. Psychol. Q. 63, 284–297. doi: 10.2307/2695840

[ref60] Susilo Y. O. Liu C. Börjesson M. (2019). The changes of activity-travel participation across gender, life-cycle, and generations in Sweden over 30 years. Transportation 46, 793–818. doi: 10.1007/s11116-018-9868-5

[ref27] Swedish Commerce (2021). Läget i handeln. 2021 års rapport om branschens ekonomiska utveckling [The situation in commerce. 2021 years report on the economic development in this branch]. Stockholm: HUI Research.

[ref45] Swedish Government Office (2020). *Cirkulär ekonomi – strategi för omställningen i Sverige.* [Circular economy – strategy for the transition in Sweden]. Stockholm: Swedish Government Office.

[ref61] Teixeira P. J. Carraça E. V. Markland D. Silva M. N. Ryan R. M. (2012). Exercise, physical activity, and self-determination theory: a systematic review. Int. J. Behav. Nutr. Phys. Act. 9:78. doi: 10.1186/1479-5868-9-78, PMID: 22726453 PMC3441783

[ref62] Testa F. Pretner G. Iovino R. Bianchi G. Tessitore S. Iraldo F. (2021). Drivers to green consumption: a systematic review. Environ. Dev. Sustain. 23, 4826–4880. doi: 10.1007/s10668-020-00844-5

[ref23] The Swedish Outdoor Association (2024). *Om oss.*[About us.]. Available at: https://svensktfriluftsliv.se/om-oss/ (Accessed January 30, 2024).

[ref49] The Swedish Sports Confederation (2021). Idrottsrörelsen i siffror. [the sports movement in numbers]. Stockholm: The Swedish Sports Confederation.

[ref48] The Swedish Sports Confederation (2024). Medlemmar. [Members.] Available at: https://idrottsstatistik.se/foreningsidrott/medlemmar/ (Accessed January 30, 2024).

[ref63] Theodori G. L. Luloff A. E. Willits F. K. (1998). The Association of Outdoor Recreation and Environmental Concern: reexamining the Dunlap-Heffernan thesis! Rural. Sociol. 63, 94–108. doi: 10.1111/j.1549-0831.1998.tb00666.x

[ref64] Thormann T. F. Wicker P. (2021). Determinants of pro-environmental behavior among voluntary sport club members. Ger. J. Exerc. Sport Res. 51, 29–38. doi: 10.1007/s12662-020-00700-8

[ref65] Timmer S. Bösehans G. Henkel S. (2023). Behavioural norms or personal gains? An empirical analysis of commuters intention to switch to multimodal mobility behaviour. Transp. Res. A Policy Pract. 170:103620. doi: 10.1016/j.tra.2023.103620

[ref66] United Nations (2015). Transforming our world: The 2023 agenda for sustainable development. New York: United Nations.

[ref67] van der Werff E. Steg L. Keizer K. (2013a). It is a moral issue: the relationship between environmental self-identity, obligation-based intrinsic motivation and pro-environmental behaviour. Glob. Environ. Change 23, 1258–1265. doi: 10.1016/j.gloenvcha.2013.07.018

[ref68] van der Werff E. Steg L. Keizer K. (2013b). The value of environmental self-identity: the relationship between biospheric values, environmental self-identity and environmental preferences, intentions and behaviour. J. Environ. Psychol. 34, 55–63. doi: 10.1016/j.jenvp.2012.12.006

[ref69] van der Werff E. Steg L. Keizer K. (2014). I am what I am, by looking past the present: the influence of Biospheric values and past behavior on environmental self-identity. Environ. Behav. 46, 626–657. doi: 10.1177/0013916512475209

[ref70] Verduzco Torres J. R. Hong J. McArthur D. P. (2022). How do psychological, habitual and built environment factors influence cycling in a city with a well connected cycling infrastructure? Int. J. Urban Sci. 26, 478–498. doi: 10.1080/12265934.2021.1930111

[ref71] Vesely S. Masson T. Chokrai P. Becker A. M. Fritsche I. Klöckner C. A.. (2021). Climate change action as a project of identity: eight meta-analyses. Glob. Environ. Change 70:102322. doi: 10.1016/j.gloenvcha.2021.102322

[ref72] Wallén Warner H. Björklund G. Andersson J. (2021). Using a three-stage model of change to understand people’s use of bicycle, public transport, and car. Trans. Res. F Traffic Psychol. Behav. 82, 167–177. doi: 10.1016/j.trf.2021.08.002

[ref73] Wang P. Y. Lyons K. Young T. (2023). Role identities of adventure tour operators in national parks. Curr. Issues Tour. 27, 3017–3029. doi: 10.1080/13683500.2023.2265033

[ref74] Whitmarsh L. E. Haggar P. Thomas M. (2018). Waste reduction behaviors at home, at work, and on holiday: what influences behavioral consistency across contexts? Front. Psychol. 9:2447. doi: 10.3389/fpsyg.2018.02447, PMID: 30574111 PMC6291483

[ref75] Whitmarsh L. O’Neill S. (2010). Green identity, green living? The role of pro-environmental self-identity in determining consistency across diverse pro-environmental behaviours. J. Environ. Psychol. 30, 305–314. doi: 10.1016/j.jenvp.2010.01.003

[ref9001] Whitmarsh L. Player L. Jiongco A. James M. Williams M. Marks E.. (2022). Climate anxiety: What predicts it and how is it related to climate action? J. Environ. Psychol. 83:101866. doi: 10.1016/j.jenvp.2022.101866

[ref76] Wicker P. (2019). The carbon footprint of active sport participants. Sport Manag. Rev. 22, 513–526. doi: 10.1016/j.smr.2018.07.001

[ref77] World Health Organization (WHO) (2020). WHO guidelines on physical activity and sedentary behaviour: At a glance. Geneva: World Health Organization.

[ref78] Yener G. Secer A. Ghazalian P. L. (2023). What factors influence consumers to buy Green products? An analysis through the motivation–opportunity–ability framework and consumer awareness. Sustain. For. 15:13872. doi: 10.3390/su151813872

