# Supplementary material for: Integrating drivers of pro-environmental behavior and physical activity to explore (in) compatibilities between an active and an environmentally sustainable lifestyle
Source: Front Psychol. 2024 Dec 11;15:1397320. doi: 10.3389/fpsyg.2024.1397320 (PMC11668964; doi:10.3389/fpsyg.2024.1397320)
Supplement: Supplementary file 1 [file Table_1.docx]

**Supplementary materials**

Table S1. Measures.

| Concept | Items |
| --- | --- |
| Environmental self-identity (ENV_IDEN) | Acting environmentally-friendly is an important part of who I am; I am the type of person who acts environmentally-friendly; I see myself as an environmentally-friendly person. Five-point scale (Completely disagree, Completely agree). |
| Autonomous environmental motivation (ENV_AUTO) Five-point scale (Not at all, Completely). | |
| Intrinsic | I am environmentally friendly because it is fun; I feel joy and satisfaction when I act environmentally friendly; I like to act environmentally friendly. |
| Introjected | I think it is important to try and act environmentally friendly; There are benefits associated with reducing my environmental impact that I value; It is important for me to act environmentally friendly regularly. |
| Controlled environmental motivation (ENV_CONT) Five-point scale (Not at all, Completely). | |
| Introjected | I feel I should reduce my environmental impact when it is possible; I am ashamed if I miss an opportunity to act environmentally friendly; I get a bad conscious if I don’t act environmentally friendly. |
| External | I act environmentally friendly because others (family or friends) say I should; I am environmentally friendly so that people in my surroundings will have a positive image of me; I feel pressured by people in my surroundings to be environmentally friendly. |
| Athlete identity (ATHLETE_IDEN) | To what extent would you describe yourself in the following ways? As an athlete. Five-point scale (Not at all, A little, Partly, A lot, Completely). |
| Outdoor identity (OUTDOOR_IDEN) | To what extent would you describe yourself in the following ways? As an outdoor person. Five-point scale (Not at all, A little, Partly, A lot, Completely). |
| Autonomous activity motivation (ACT_AUTO) Five-point scale (Not at all, Completely). | |
| Intrinsic | I am physically active because it is fun; I feel joy and satisfaction when I am physically active; I like to be physically active. |
| Introjected | I think it is important to try and be physically active; There are benefits associated with being physically active that I value; It is important for me to be physically active regularly. |
| Controlled activity motivation (ACT_CONT) Five-point scale (Not at all, Completely). | |
| Introjected | I feel I should be physically active when it is possible; I am ashamed if I miss an opportunity to be physically active; I get a bad conscious if I am not physically active. |
| External | I am physically active because others (family or friends) say I should; I am physically active so that people in my surroundings will have a positive image of me; I feel pressured by people in my surroundings to be physically active. |
| Physical activity environmentally significant behaviors | Travel alone by car, Use public transportation (e.g., train, bus), Use bicycle or walk as a means of transport (also e-bike and electric scooter). Five-point scale (Very seldom or never, Approx. 1 times/month, Approx. 2-3 times/month, 1-2 times/week, 3-4 times/week, 5 times/week or more). Buy used equipment for physical activity in secondhand stores, Facebook, market place, online etc. Sell, exchange or donate used equipment for physical activity to secondhand stores, Facebook, market place, online etc., Buy new equipment for physical activity (both for you and others in the household). Four-point scale (Never, One or some times/year, Approx. one time/month, Several times/month). |
| Involvement in physical activity (ACT_HOUR) | How many hours on average in one week, do you participate in moderately strenuous activities such as walking? and How many hours on average in one week, do you participate in very strenuous activities such as jogging? Four-point scale (0 hours, 1-4 hours, 5-10 hours, 11 hours or more). |
| General pro-environmental behavior frequency (PEB) | Eat ecological food, locally produced or food in season, Eat red meet (e.g., beef, pork, lamb) (R), Recycle food waste, Recycle household waste (e.g., glass, plastic, metal), Travel alone by car (R), Use public transportation (e.g., train, bus), Use bicycle or walk as a means of transport (also e-bike and electric scooter). Five-point scale (Not at all, 1-2 times, 3-4 times, 5-6 times, At least one time/day). |
| Athlete activity frequency (ATHLETE_FREQ) | Engaging in athlete activities or exercise yourself (e.g., run, cycle, swim etc.), Athlete activities in a team (e.g., soccer, floorball), Downhill or cross-country skiing. Five-point scale (Every day or several times/week, Several times/month, Approx. 1 time/month, One or some times/year, More seldom or never). |
| Outdoor activity frequency (OUTDOOR_FREQ) | Spending time in forest and land, Hunting wildlife or fishing, and Birdwatching. Five-point scale (Every day or several times/week, Several times/month, Approx. 1 time/month, One or some times/year, More seldom or never). |

R = Reversed scale before computing index.
