# Supplementary material for: Integrating drivers of pro-environmental behavior and physical activity to explore (in) compatibilities between an active and an environmentally sustainable lifestyle
Source: Front Psychol. 2024 Dec 11;15:1397320. doi: 10.3389/fpsyg.2024.1397320 (PMC11668964; doi:10.3389/fpsyg.2024.1397320)
Supplement: Supplementary file 2 [file Table_2.docx]

**Supplementary materials**

Table S2. Significant effects in the model examining the impact of environmental drivers and physical activity drivers on travel mode choice and consumption associated with physical activity, while controlling for hours of physical activity, with outdoor identity as the second moderator.

| CAR_ALONE  Pseudo R^2^ _N_ = .11  -2LL = 1289.87 | ENV_AUTO (-0.25(0.11), z = -2.30 (p = .022, 95% CIs [-0.47 -0.04])  ACT_CONT (0.32(0.12), z = 2.60 (p = .009, 95% CIs [0.08 0.56])  ATHLETE_IDEN (0.39(0.07), z = 5.36 (p < .001, 95% CIs [0.25 0.53])  OUTDOOR_IDEN (0.18(0.07), z = 2.77 (p < .001, 95% CIs [0.05 0.31])  ENV_IDEN*ACT_AUTO (-0.47(0.15), z = -3.21 (p < .001, 95% CIs [-0.77 -0.19]) |
| --- | --- |
| PUB  Pseudo R^2^ _N_ = .10  -2LL = 1154.83 | ACT_CONT (0.59(0.13), z = 4.63 (p < .001, 95% CIs [0.34 0.84]) |
| CYCLE/WALK  Pseudo R^2^ _N_= .10  -2LL = 1142.39 | ACT_AUTO (0.34(0.10), z = 3.41 (p < .001, 95% CIs [0.15 0.54])  ACT_HOUR (0.42(0.16), z = 2.64 (p < .001, 95% CIs [0.11 0.73]) |
| Pseudo R^2^ _N_ | ACT_AUTO (0.63(0.14), z = 4.67 (p < .001, 95% CIs [0.37 0.90])  ATHLETE_IDEN (0.39(0.15), z = 2.56 (p = .010, 95% CIs [0.09 0.68]) |
| BUY_USED  Pseudo R^2^ _N_= .16  -2LL = 1198.01 | ENV_CONT (0.55(0.14), z = 3.95 (p < .001, 95% CIs [0.28 0.83])  ACT_CONT (0.26(0.13), z = 2.05 (p = .040, 95% CIs [0.01 0.50])  OUTDOOR_IDEN (0.36(0.07), z = 5.08 (p < .001, 95% CIs [0.22 0.50])  ENV_AUTO*ACT_AUTO (0.24(0.12), z = 1.97 (p = .049, 95% CIs [0.00 0.49])  ENV_CONT*OUTDOOR_IDEN (-0.28(0.11), z = -2.54 (p = .011, 95% CIs [-0.50 -0.06]) |
| SELL_USED  Pseudo R^2^ _N_= .12  -2LL = 1282.82 | ENV_CONT (0.29(0.13), z = 2.20 (p = .028, 95% CIs [0.03 0.55])  OUTDOOR_IDEN (0.21(0.07), z = 3.16 (p = .002, 95% CIs [0.08 0.34])  ATHLETE_IDEN (0.16(0.07), z = 2.28 (p = .023, 95% CIs [0.02 0.30]) |

Environmental self-identity (ENV_IDEN), Environmental autonomous motivation (ENV_AUTO), Environmental controlled motivation (ENV_CONT), Activity autonomous motivation (ACT_AUTO), Activity controlled motivation (ACT_CONT), Athlete identity (ATHLETE_IDEN), Outdoor identity (OUTDOOR_IDEN), Physical activity involvement (ACT_HOUR), Use of car alone on trips to physical activity (CAR_ALONE), Use of public transport on trips to physical activity (PUB), Use of cycle/walk on trips to physical activity (CYCLE/WALK), Buy new material in relation to physical activity (BUY_NEW), Buy used material in relation to physical activity (BUY_USED), Sell used material in relation to physical activity (SELL_USED).
