# Supplementary material for: Integrating drivers of pro-environmental behavior and physical activity to explore (in) compatibilities between an active and an environmentally sustainable lifestyle
Source: Front Psychol. 2024 Dec 11;15:1397320. doi: 10.3389/fpsyg.2024.1397320 (PMC11668964; doi:10.3389/fpsyg.2024.1397320)
Supplement: Supplementary file 3 [file Table_3.docx]

**Supplementary materials**

Table S3. Bivariate correlations between drivers and pro-environmental behaviors, frequency of engaging in athlete and outdoor activities, and environmentally significant behaviors associated with physical activity.

|  | ENV_IDEN | ENV_AUTO | ENV_CONT | ACT_AUTO | ACT_CONT | ATHLETE_IDEN | OUTDOOR_IDEN |
| --- | --- | --- | --- | --- | --- | --- | --- |
| ENV_IDEN |  |  |  |  |  |  |  |
| ENV_AUTO | **.56***** |  |  |  |  |  |  |
| ENV_CONT | **.53***** | **.54***** |  |  |  |  |  |
| ACT_AUTO | .19*** | .38*** | .16*** |  |  |  |  |
| ACT_CONT | .24*** | .25*** | **.54***** | .31*** |  |  |  |
| ATHLETE_IDEN | .10** | .06 | .11*** | .39*** | .24*** |  |  |
| OUTDOOR_IDEN | .21*** | .25*** | .10*** | .34*** | .03 | .26*** |  |
| PEB | .29*** | .41*** | .24*** | .25*** | .13*** | .04 | .14*** |
| ATHLETE_FREQ | .03 | .03 | .03 | .33*** | .14** | .41*** | .17*** |
| OUTDOOR_FREQ | .00 | -.01 | -.08** | .05 | -.08* | .10** | .36*** |
| ACTIVITY_HOUR | .11*** | .13*** | .06* | .41*** | .14*** | .42*** | .30*** |
| CAR_ALONE | -.05 | -.08* | .02 | .08* | .11** | .22*** | .10*** |
| PUB | .10** | .07* | .16*** | .03 | .22*** | .10*** | -.02 |
| CYCLE/WALK | .09** | .08* | .09** | .22*** | .11*** | .17*** | .09** |
| BUY_USED | .17*** | .16*** | .23*** | .14*** | .18*** | .12*** | .21*** |
| SELL_USED | .16*** | .15*** | .17*** | .19*** | .14*** | .17*** | .18*** |
| BUY_NEW | .11*** | .12*** | .12*** | .35*** | .19*** | .20*** | .13*** |

*p < .05. **p < .01, ***p < .001. Environmental self-identity (ENV_IDEN), Environmental autonomous motivation (ENV_AUTO), Environmental controlled motivation (ENV_CONT), Activity autonomous motivation (ACT_AUTO), Activity controlled motivation (ACT_CONT), Athlete identity (ATHLETE_IDEN), Outdoor identity (OUTDOOR_IDEN), Pro-environmental behaviors (PEB), Frequency of athlete activities (ATHLETE_FREQ), Frequency of outdoor activities (OUTDOOR_FREQ), Physical activity involvement (ACT_HOUR), Use of car alone on trips to physical activity (CAR_ALONE), Use of public transport on trips to physical activity (PUB), Use of cycle/walk on trips to physical activity (CYCLE/WALK), Buy used material in relation to physical activity (BUY_USED), Sell used material in relation to physical activity (SELL_USED), Buy new material in relation to physical activity (BUY_NEW). Pearson *r* for all variables except environmentally significant behaviors associated with physical activity for which Point biserial correlation is used. Numbers in grey indicates a small effect size (0.1-0.3), in black, a medium effect size (0.3-0.5), and in bold, a large effect size (>0.5), according to Cohen, J. (1988). Statistical power analysis for the behavioral sciences (2nd ed.). New York: Routledge.
