# Supplementary material for: Integrating drivers of pro-environmental behavior and physical activity to explore (in) compatibilities between an active and an environmentally sustainable lifestyle
Source: Front Psychol. 2024 Dec 11;15:1397320. doi: 10.3389/fpsyg.2024.1397320 (PMC11668964; doi:10.3389/fpsyg.2024.1397320)
Supplement: Supplementary file 4 [file Table_4.docx]

**Supplementary materials**

Table S4. Overview of support for hypotheses 1-15 in relation to the different environmentally significant behaviors.

|  | Travel mode choice | | | Consumption | | |
| --- | --- | --- | --- | --- | --- | --- |
|  | Car use alone | Public transport | Cycle/walk | Buy new | Buy used | Sell used |
| **Environmental drivers** |  |  |  |  |  |  |
| **H1** ENV_IDEN ENV _AUTO | Supported | Supported | Supported | Supported | Supported | Supported |
| **H2** ENV_IDEN ENV _CONT | Supported | Supported | Supported | Supported | Supported | Supported |
| **H3** ENV_IDEN |  |  |  |  |  |  |
| **H4** ENV_AUTO | Supported |  |  |  |  |  |
| **H5** ENV_CONT |  |  |  |  | Supported | Supported |
| **Physical activity drivers** |  |  |  |  |  |  |
| **H6** ACT_AUTO |  |  | Supported | Supported |  |  |
| **H7** ACT_CONT | Supported | Supported |  |  | Supported |  |
| **H8** ATHLETE_IDEN | Supported |  |  | Supported |  | Supported |
| **H9** OUTDOOR_IDEN | Supported |  |  |  | Supported | Supported |
| **Interactions** |  |  |  |  |  |  |
| **H10** ENV_IDEN * ACT AUTO | Supported |  |  |  | Supported |  |
| **H11** ENV_AUTO* ACT_AUTO |  |  |  |  |  |  |
| **H12** ENV_CONT* ACT_AUTO |  |  |  |  |  |  |
| **H13** ENV_IDEN* ATHLETE_IDEN |  |  |  |  |  |  |
| **H14** ENV_AUTO* ATHLETE_IDEN |  |  |  |  | Supported |  |
| **H15** ENV_CONT* ATHLETE_IDEN |  |  |  |  |  |  |

Environmental self-identity (ENV_IDEN), Environmental autonomous motivation (ENV_AUTO), Environmental controlled

motivation (ENV_CONT), Activity autonomous motivation (ACT_AUTO), Activity controlled motivation (ACT_CONT), Athlete

identity (ATHLETE_IDEN), Outdoor identity (OUTDOOR_IDEN), Use of car alone on trips to physical activity (CAR_ALONE),

Use of public transport on trips to physical activity (PUB), Use of cycle/walk on trips to physical activity (CYCLE/WALK), Buy used

material in relation to physical activity (BUY_USED), Sell used material in relation to physical activity (SELL_USED), Buy new

material in relation to physical activity (BUY_NEW).
